# Prognostic value of baseline and early response FDG-PET/CT in patients with refractory and relapsed aggressive B-cell lymphoma undergoing CAR-T cell therapy

**DOI:** 10.1007/s00432-023-04587-4

**Published:** 2023-01-20

**Authors:** Thomas Walter Georgi, Lars Kurch, Georg-Nikolaus Franke, Madlen Jentzsch, Sebastian Schwind, Carmen Perez-Fernandez, Naima Petermann, Maximilian Merz, Klaus Metzeler, Gudrun Borte, Sandra Hoffmann, Marco Herling, Timm Denecke, Regine Kluge, Osama Sabri, Uwe Platzbecker, Vladan Vučinić

**Affiliations:** 1 Department of Nuclear Medicine, University of Leipzig, Leipzig, Germany; 2 Medical Clinic I, Department of Hematology, Cellular Therapy and Hemostaseology, University of Leipzig, Liebigstr. 22, 04103 Leipzig, Germany; 3 Department of Radiology, University of Leipzig, Leipzig, Germany

**Keywords:** CAR-T cells, Aggressive lymphoma, FDG-PET/CT, Cell therapy

## Abstract

**Purpose:**

Chimeric antigen receptor (CAR)-T cells are a viable treatment option for patients with relapsed or refractory (r/r) aggressive B-cell lymphomas. The prognosis of patients who relapse after CAR-T cell treatment is dismal and factors predicting outcomes need to be identified. Our aim was to assess the value of FDG-PET/CT in terms of predicting patient outcomes.

**Methods:**

Twenty-two patients with r/r B-cell lymphoma who received CAR-T cell treatment with tisagenlecleucel (*n* = 17) or axicabtagene ciloleucel (*n* = 5) underwent quantitative FDG-PET/CT before (PET-0) and 1 month after infusion of CAR-T cells (PET-1). PET-1 was classified as complete metabolic response (CMR, Deauville score 1–3) or non-CMR (Deauville score 4–5).

**Results:**

At the time of PET-1, 12/22 (55%) patients showed CMR, ten (45%) patients non-CMR. 7/12 (58%) CMR patients relapsed after a median of 223 days, three of them (25%) died. 9/10 (90%) non-CMR patients developed relapse or progressive disease after a median of 91 days, eight of them (80%) died. CMR patients demonstrated a significantly lower median total metabolic tumor volume (TMTV) in PET-0 (1 ml) than non-CMR patients (225 ml).

**Conclusion:**

Our results confirm the prognostic value of PET-1. 42% of all CMR patients are still in remission 1 year after CAR T-cell treatment. 90% of the non-CMR patients relapsed, indicating the need for early intervention. Higher TMTV before CAR-T cell infusion was associated with lower chances of CMR.

**Supplementary Information:**

The online version contains supplementary material available at 10.1007/s00432-023-04587-4.

## Introduction

Chimeric antigen receptor (CAR)-T cells are an effective new option for the treatment of patients with refractory or relapsed (r/r) aggressive B-cell lymphoma (Vucinic et al. 2021). Currently, three products, namely tisagenlecleucel, axicabtagene ciloleucel, and lisocabtagene maraleucel are licensed for the treatment of aggressive B-cell lymphoma in the European Union (Schuster et al. 2017, 2019; Neelapu et al. 2017; Locke et al. 2019; Abramson et al. 2020).

^18^F-Fluorodeoxyglucose positron emission tomography (FDG-PET)/computed tomography (CT) is the most informative diagnostic method for lymphoma imaging and has a key role in tailoring individual treatment strategies (Meignan et al. 2009; Barrington and Kluge 2017). FDG-PET/CT scans prior, during, and after treatment are useful for an appropriate treatment intensity and an early detection of relapse (Nanni et al. 2017; Mauz-Körholz et al. 2022). In addition to a purely visual assessment, quantitative parameters like the tracer uptake of the most active lymphoma lesions or the total metabolic tumor volume (TMTV) have been shown to be predictive for patients’ outcome (Hasenclever et al. 2014; Dean et al. 2020; Kurch et al. 2021). FDG-PET/CT imaging was also the method of choice for response evaluation in the pivotal clinical trials with CAR-T cells (Schuster et al. 2017, 2019; Neelapu et al. 2017; Locke et al. 2019; Abramson et al. 2020).

CAR-T cell therapy for r/r aggressive B-cell lymphomas was first approved in Europe in 2018. Until the end of 2021, 702 patients with r/r lymphoma underwent CAR-T cell treatment in Germany (DRST Annual Report 2021).

Currently, there are no standardized guidelines for imaging-based follow-up of lymphoma patients after CAR-T cell therapy. Response assessment by FDG-PET/CT is often not reimbursed by health insurances in a real-world setting because of limited available evidence. This emphasizes the need for an analysis of FDG-PET/CT scans regarding the assessment of early response to CAR-T cell therapy and the derivation of prognostic factors.

Published reports on FDG-PET/CT-based imaging in the context of CAR-T cell treatment mostly focused on scans before (PET-0) and 1 month after (PET-1) the infusion of CAR-T cells (Shah et al. 2018; Dean et al. 2020; Cohen et al. 2021; Derlin et al. 2021; Vercellino et al. 2021; Sesques et al. 2021; Hong et al. 2021; Iacoboni et al. 2021; Breen et al. 2022; Reinert et al. 2022; Al Zaki et al. 2022). In some of these publications, lymphoma response in PET-1 seems to be predictive for progression-free survival (PFS) and overall survival (OS) (Shah et al. 2018; Cohen et al. 2021; Iacoboni et al. 2021; Al Zaki et al. 2022). However, the results concerning the prognostic value of quantitative PET parameters were ambiguous. Significant correlations between TMTV in PET-0 and clinical outcome after CAR-T cell treatment were reported (Bishop et al. 2019; Dean et al. 2020; Derlin et al. 2021; Vercellino et al. 2021; Breen et al. 2022; Reinert et al. 2022), while other authors could not confirm this association (Wang et al. 2019; Sesques et al. 2021). The maximum standard uptake value (SUVmax) of the most active residual lymphoma lesion in PET-1 might also be predictive for outcomes (Sesques et al. 2021; Breen et al. 2022; Al Zaki et al. 2022).

The aim of our analysis was the evaluation of FDG-PET/CT imaging in patients with r/r B-cell lymphoma undergoing CAR-T cell treatment in our academic center in a real-world setting and based on our institutional guidelines. We focused on the prognostic value of PET-1 response and on the predictive power of quantified PET parameters with respect to relevant clinical outcomes.

## Patients and methods

### Study design

We retrospectively analyzed 22 adult patients with r/r aggressive B-cell lymphoma undergoing treatment with CAR-T cells between 20th May 2019 and 14th September 2021, including standardized PET/CT imaging at the Medical Center of the University of Leipzig. Only the patients who underwent PET-1 examination were included in the analysis. The median patient age at the infusion of CAR-T cells was 62 years (range 18–73). 16 patients were male (73%), six were female (27%). 16 patients (73%) were treated with tisagenlecleucel (Kymriah^®^) and six patients (27%) received axicabtagene ciloleucel (Yescarta^®^). Median lactate dehydrogenase (LDH) was 277 U/l (range 198–1542). Most patients (87%) had LDH levels increased above the upper level of normal (ULN) (213 U/l). Lymphodepleting chemotherapy was applied on day-5, -4 and -3 prior to infusion of CAR-T cells and consisted of 30 mg/m^2^ fludarabine and 500 mg/m^2^ cyclophosphamide per day for patients treated with axicabtagene ciloleucel, and 25 mg/m^2^ fludarabine and 250 mg/m^2^ cyclophosphamide for patients treated with tisagenlecleucel. Fifteen patients had been diagnosed with a diffuse large B-cell lymphoma (DLBCL) (68%), five patients with transformed follicular lymphoma (tFL) (22%), and two patients with primary mediastinal B-cell lymphoma (PMBCL) (9%). Patients were treated after median of four previous treatment lines (range 2–6). Five patients had bone marrow infiltration. The median follow-up after infusion of CAR-T cells was 297 days (range 28–864). Patients’ characteristics are presented in Table 1.

**Table 1:** Demographic characteristics of patients

Characteristics	
Patients, *n*	22
Male (%)	16 (72.7)
Female (%)	6 (27.3)
Diagnosis	
DLBCL (%)	15 (68.2)
tFL (%)	5 (22.7)
PMBCL (%)	2 (9.1)
Reinfused product	
Tisagenlecleucel (%)	17 (77.3)
Axicabtagene ciloleucel (%)	5 (22.7)
Prior treatment lines, median (range)	4 (2–6)
bone marrow infiltration, *n* (%)	17 (77.3)
LDH at LD, median (range)	277 (198–1542)
≤ ULN (213 U/l) (%)	3 (13.6)
> ULN (213 U/l) (%)	19 (86.4)

*DLBCL* diffuse large B-cell lymphoma, *tFL* transformed follicular lymphoma, *PMBCL* primary mediastinal B-cell lymphoma, *LDH* lactate dehydrogenase, *LD* lymphodepletion, *ULN* upper level of normal

### FDG-PET/CT imaging

An FDG-PET/CT scan before start of treatment was performed in 18 of 22 patients. All 22 patients had an FDG-PET/CT scan 1 month after CAR-T cell treatment.

### FDG-PET/CT assessment

All FDG-PET/CT scans were assessed by two nuclear physicians and two radiologists. PET images were assessed visually according to the Deauville score (DS) (Cheson et al. 2014) and classified as complete metabolic response (CMR) with DS 1–3, partial metabolic response (PMR) with DS 4–5, or progressive disease (PD) (Ferrari et al. 2021). Additionally, lymphoma lesions were assessed quantitatively using the Hybrid3D viewer software (HERMES Medical Solutions) as previously reported (Georgi et al. 2022). TMTV was measured using an SUV of 3.0 to define an outer boundary around the lymphoma lesions. In few cases, manual adjustments were necessary for an optimal tumor delineation. SUVmax and the mean standard uptake value (SUVmean) of the lymphoma lesions were measured, and the total lesion glycolysis (TLG) was calculated as TLG = SUVmean * TMTV. The morphological tumor volume was measured in CT according to the recommendation of the Lugano classification. (Cheson et al. 2014).

### Statistical analysis

OS was defined as survival from the time of infusion of CAR-T cells, regardless of disease status. Surviving patients were censored at last follow-up, and only death was considered as event. PFS was defined as the time from infusion of CAR-T cells to relapse, disease progression, or death from any cause. Two patients underwent the successive treatment with allogeneic HSCT and were censored for further analyses at day of PFS and OS were assessed according to the Kaplan–Meier estimate and groups were compared using the Log-rank test. Non-relapse-mortality (NRM) was defined as the probability of death from any cause other than recurrence of the disease after the infusion of CAR-T cells. NRM and cumulative incidence of relapse or progression were considered to be reciprocal competing risks and were calculated according to the Fine and Gray model. (Fine and Gray 1999) Curve comparisons and *p* values were analyzed using R (version 3.6.2) (R Core Team 2020).

## Results

### Baseline imaging

The median time between PET-0 and infusion of CAR-T cells was 6 days. Fifteen out of 18 patients with PET-0 imaging showed metabolically active lymphoma tissue, with DS 4 in five patients and DS 5 in ten. The other three patients had a CMR with DS 1 in two patients and DS 2 in one. Twelve patients underwent bridging therapy prior to reinfusion of CAR-T cells, five (42%) with chemotherapy, six (50%) with antibody conjugates and one (8%) revlimid based. Only one patient achieved a CMR after the revlimid-based bridging therapy.

### Early response assessment (PET-1)

The median time between the CAR-T cell treatment and PET-1 was 29 days. Twelve of 22 patients (54%) showed CMR in PET-1, nine patients (41%) PMR and one patient (5%) PD with an increase in lymphoma volume and tumor activity. Among the twelve patients with CMR in PET-1, five patients had DS 1, one DS 2 and six DS 3. Among the nine patients with PMR, five patients had DS 4 and four DS 5.

Out of the 15 patients with active lymphoma tissue in PET-0, eight converted to CMR in PET-1 as a result of CAR-T cell therapy, six showed PMR and one PD. The three patients without active lymphoma tissue in PET-0 were all still in CMR in PET-1. The morphologic tumor response of patients with CMR showed complete response (CR) in five patients, partial response (PR) in four patients and stable disease (SD) in two patients. In the non-CMR patients, morphological PR was observed in four patients, SD in two patients and PD in one patient.

### Clinical outcomes

**Fig. 1:**
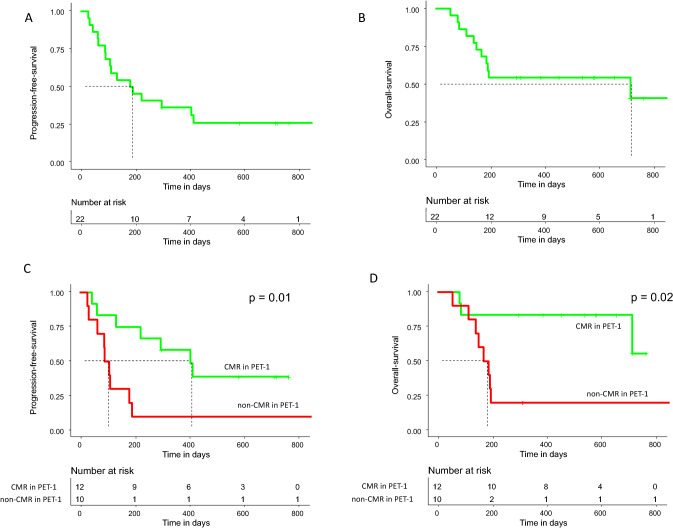
**A** Progression free survival (PFS) of all patients. **B** Overall survival (OS) of all patients. **C** PFS of patients with complete metabolic response (CMR) and without CMR (non-CMR) in PET-1. **D** OS of CMR and non-CMR patients in PET-1

The median PFS of all patients was 187 days, and the median OS was 717 days. After 1 year, 36% of all patients were still in CMR and 54% were still alive. None of the patients died of NRM causes (Fig. 1A + B). Patients with CMR in PET-1 showed superior outcomes with a median PFS of 407 days and a median OS that was not reached, compared to non-CMR (PMR and PD) patients with a median PFS of 100 days (*p* = 0.01) and a median OS of 178 days (*p* = 0.02) (Fig. 1C + D). Furthermore, the PFS and the OS of patients with morphological CR or PR were also better than that of patients with SD or PD (see Supplemental material).

Seven of twelve (58%) patients with CMR in PET-1 relapsed after a median of 223 days (range 45–415). Three of them died (25%), 81, 563 and 717 days after reinfusion of CAR-T cells, respectively. Of the remaining four relapsed patients, one underwent allogeneic hematopoietic stem cell transplantation (allo-HSCT) at 392 days after reinfusion of CAR-T cells. Two were treated with tafasitamab/lenalidomide, 459 and 545 days after CAR-T cell treatment, and one patient received radiotherapy and has stable disease 415 days after CAR-T cell reinfusion.

Nine of ten non-CMR patients developed relapse or progressive disease after a median of 91 days (range 28–192) post infusion of CAR-T cells, and eight of them died. One patient is alive after allo-HSCT, performed 318 days after CAR-T cell treatment. Only one non-CMR patient in PET-1 achieved CMR in a subsequent FDG-PET/CT 6 months after CAR-T cell therapy without further treatment. This patient is still in remission.

**Fig. 2:**
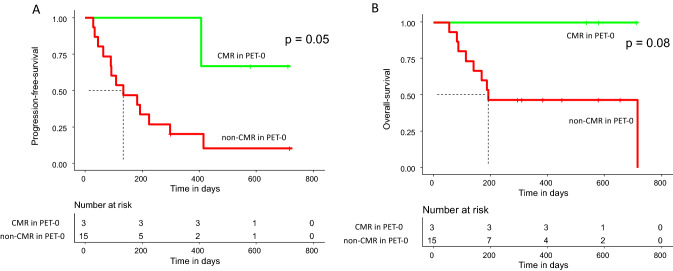
**A** Progression free survival of patients with complete metabolic response (CMR) and without CMR (non-CMR) in PET-0. **B** Overall survival of patients CMR and non-CMR patients in PET-0

The three patients without metabolically active lymphoma lesions in PET-0 showed superior PFS when compared to the 15 patients with vital lymphoma lesions in PET-0 (*p* = 0.05). Two of them were still in CMR at last follow-up, one patient relapsed after 407 days and received further treatment with tafasitamab/lenalidomide. All three patients were alive 402, 587, and 720 days after infusion of CAR-T cells, respectively (Fig. 2).

Out of the 15 patients with metabolically active tumor tissue in PET-0, eight patients achieved CMR in PET-1 and seven non-CMR. Out of the eight CMR patients, six (75%) relapsed and three (38%) died. Out of the seven non-CMR patients, all of them relapsed, and six (86%) died.

### Assessment of potentially prognostic factors

The median pre-treatment LDH in patients with CMR in PET-1 was 250 U/l (range 199–726) and 386 U/l (range 245–1297) in non-CMR patients (*p* = 0.01).

**Fig. 3:**
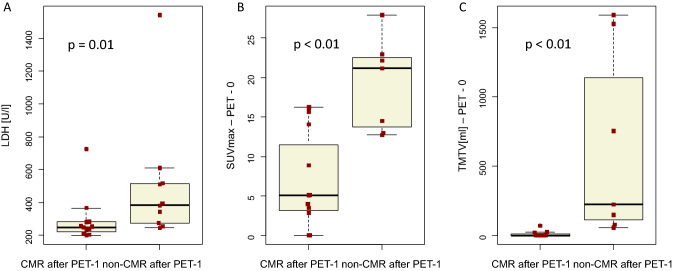
Lactate dehydrogenase at lymphodepletion (**A**) and quantitative parameter in PET-0 (maximum standard uptake value (SUVmax) (**B**) and total metabolic tumor volume (TMTV) (**C**)) in patients with complete metabolic response (CMR) and without CMR (non-CMR) in PET-1

Quantitative PET parameters are presented in Table 2. The twelve patients with CMR in PET-1 showed significantly lower values for SUVmax (median 5.1; range 3.5–16.2) and for TMTV (median 1 ml; range 1–71 ml) in their PET-0 than the ten patients with non-CMR, which showed a median SUVmax of 21.2 (range 12.7–27.9) and a median TMTV of 225 ml (range 56–1592 ml), (*p* < 0.01, respectively; Fig. 3).

**Table 2:** Quantitative PET parameter before (PET-0) and 1 month after CAR-T cell therapy (PET-1)

Quantitative PET parameter	Median (range)
PET-0	
SUVmax	12.9 (0.0–27.9)
SUVmean	4.3 (0.0–10.7)
TMTV [ml]	23 (0–1592)
TLG [ml]	112 (0–8041)
PET-1	
SUVmax	4.1 (0.0–31.4)
SUVmean	3.4 (0.0–9.7)
TMTV [ml]	2 (0–262)
TLG [ml]	5 (0–2544)

*SUVmax* maximum standard uptake value, *SUVmean* mean standard uptake value, *TMTV* total metabolic tumor volume, *TLG* total lesion glycolysis

Based on our TMTV measurements, a value of 25 ml in PET-0 suits well for an optimal discrimination between patients with CMR and non-CMR in PET-1. All patients with a TMTV ≤ 25 ml in PET-0 achieved CMR in PET-1, whereas only one patient with a TMTV > 25 ml showed CMR in PET-1.

In PET-1, non-CMR patients showed a median SUVmax of 7.7 (range 4.5–31.4) and a median TMTV of 24 ml (range 2–262 ml). Compared to PET-0, this corresponded to an average reduction of 64% of the median SUVmax, and of 90% of the median TMTV. Two of five non-CMR patients with a TMTV ≤ 25 ml in PET-1 were still alive at last follow-up, whereas all 5 patients with a TMTV > 25 ml in PET-1 had died.

## Discussion

The results of our analysis confirm the prognostic value of FDG-PET/CT imaging early after CAR-T cell treatment of patients with r/r aggressive B-cell lymphoma. Patients who achieved CMR at PET-1 showed significantly better outcomes than non-CMR patients. One year after infusion of CAR-T cells, 42% of CMR patients maintained their remission without further treatment. In contrast, 90% of non-CMR patients relapsed within 1 year after CAR-T cell therapy and 80% of them died. Our results are in line with published data concerning the predictive value of lymphoma responses in FDG-PET/CT 1 month after CAR-T cell treatment for PFS and OS (Shah et al. 2018; Cohen et al. 2021; Iacoboni et al. 2021; Al Zaki et al. 2022).

Pseudoprogression after CAR-T cell infusion has been described in a few reports (Wang et al. 2019; Boursier et al. 2022) and is defined as temporary appearance of new lesions or increase in existing lesions without clinical deterioration, due to inflammatory changes under immunomodulatory treatment (Cheson et al. 2016). Pseudoprogression might affect early FDG-PET response assessment but was not observed in our patients.

The prognosis of lymphoma relapse after treatment with CAR-T cells is dismal with very limited treatment options. There is no standard of care for salvage therapies after CAR-T cells. Spiegel et al. reported about salvage strategies in relapsed patients after axicabtagene ciloleucel in a real-world setting (Spiegel et al. 2020). Of their 135 evaluable patients, 26% received best supportive care. Among the patients undergoing treatment, only eight patients (6%) proceeded to allo-HSCT, with three of them achieving lasting remissions. These poor results reflect patient-related issues such as comorbidities or toxicities of previous therapies, but also the limitations in donor availability and the current lack of effective salvage options. However, the detection of imminent relapse, e.g., non-CMR in PET-1, enables early intervention and might increase the success of salvage therapies.

The TMTV in PET-0 seems to be predictive for the PET-1 response. Patients achieving CMR in PET-1 had a significant lower pre-treatment median TMTV compared to non-CMR patients. The prognostic value of TMTV in PET-0 concerning the clinical outcome after CAR-T cell treatment was also reported by other authors (Dean et al. 2020; Derlin et al. 2021; Vercellino et al. 2021; Iacoboni et al. 2021; Breen et al. 2022; Reinert et al. 2022). Despite the fact that Wang et al. (2019) could not confirm a significant difference in the median baseline TMTV between responder and non-responder to CAR-T cell therapy, responder showed a clearly smaller volume of 58 ml compared to non-responder with 111 ml. Sesques et al. (2021) were not able to show a statistically significant difference in patients outcomes based on baseline TMTV. However, they reported about the predictive value of the metabolic volume kinetics before CAR-T cell therapy for PFS, namely the increase of the TMTV between leukapheresis and infusion of CAR-T cells. The specification of a universal threshold value for the TMTV in PET-0 is not possible since the TMTV measurement is not uniform. Some authors used relative thresholds of 41%, 45% or 50% of the SUVmax for tumor delineation (Dean et al. 2020; Derlin et al. 2021; Reinert et al. 2022) Other authors preferred a fixed threshold of an SUVmax of 2.5 with manual modifications (Breen et al. 2022).

The SUVmax in PET-0 and the pre-infusion LDH level seem to be also predictive for early FDG-PET response. CMR patients in our study demonstrated a clearly lower median SUVmax of 5.1 in PET-0 than non-CMR patients with 21.2 (*p* < 0.01). Elevated pre-infusion LDH values indicating disease activity were reported to be associated with worse outcome (Hirayama et al. 2019; Neelapu 2019; Vercellino et al. 2020). Our data confirm this result, showing significantly lower LDH values in patients who responded CAR-T cell treatment.

In summary, our data show that lower baseline tumor burden is associated with better outcome after CAR-T cell treatment, thus putting the role of bridging therapy in focus, with its goal to achieve the maximum reduction of tumor burden before infusion. Our results and those published by other groups raise the question, if the goal of bridging should be to achieve CMR in PET-0. In accordance with this hypothesis, Bishop et al. reported about a subpopulation of seven patients treated with tisagenlecleucel in the JULIET trial who had no measurable disease in PET-0. In the PET/CT scan after 3 months, all seven patients were in CMR, and five showed persistent CMR 1 year after treatment (Bishop et al. 2019). In our analysis, three patients showed CMR before lymphodepletion, two of them are still in persistent CMR, 587 and 720 days after the infusion, respectively. Although not allowed in the pivotal ZUMA-1 trial, most patients in real-world analyses underwent bridging therapies before CAR-T cell reinfusion. In a US real-world analysis of 275 patients treated with axicabtagene ciloleucel, 53% were bridged with chemotherapy, targeted therapies, corticosteroids or radiation (Nastoupil et al. 2020). The proportion of patients undergoing bridging therapies was even higher in European series and ranged between 78 and 83% (Bethge et al. 2022; Kwon et al. 2022; Bachy et al. 2022).

Although limited by the relatively small cohort of 22 patients and the retrospective character, our study demonstrates the prognostic relevance of FDG-PET/CT imaging before and 1 month after CAR-T cell therapy and emphasizes their role in the intervention strategies after treatment.

## Supplementary Information

Below is the link to the electronic supplementary material.

Supplementary file1 (DOCX 80 kb)

## Data Availability

The data that support the findings of this study are available from corresponding author upon reasonable request.
