# Supplementary material for: Prognostic value of baseline and early response FDG-PET/CT in patients with refractory and relapsed aggressive B-cell lymphoma undergoing CAR-T cell therapy
Source: J Cancer Res Clin Oncol. 2023 Jan 20;149(9):6131–8. doi: 10.1007/s00432-023-04587-4 (PMC10356653; doi:10.1007/s00432-023-04587-4)
Supplement: Supplementary file 1 — Supplementary file1 (DOCX 80 kb) [file 432_2023_4587_MOESM1_ESM.docx]

**Supplemental material: Prognostic value of baseline and early response FDG-PET/CT in patients with refractory and relapsed aggressive B-cell lymphoma undergoing CAR-T cell therapy**

Georgi TW, Kurch L, Franke GN et al.

**Morphological assessment according to computer tomography**

One month after the reinfusion of chimeric antigen receptor (CAR)-T cells, 18 patients were evaluable for morphological assessment per computer tomography (CT). 13 patients showed complete (CR) or partial remission (PR) and five stable disease or progressive disease. The median progression-free survival (PFS) of patients with CR/PR was superior to non-CR/PR with 297 and 90 days, respectively (p<0.01). The median overall survival in CR/PR group showed a trend comparing the non-CR/PR group (p=0.07) with 717 and 169 days respectively (s. SM Figure 1)

Four patients with CR/PR in CT showed metabolic activity in FDG-PET. In two patients with SD in CT no metabolic activity was seen in FDG-PET, indicating the necessity of combining both imaging methods.

Figure SM1: Outcomes of patients with CR/PR and without CR/PR one month after reinfusion of CAR-T cells. A. Progression-free-survival. B. Overall-survival

**Outcomes of patients with metabolic CR according to the Deauville Score (DS)**

Twelve patients showed metabolic CR in PET one month after reinfusion of CAR-T cells (PET-1). The median PFS of patients with DS 3 and DS <3 was 407 and 319 days respectively (p=0.63). The OS of patients with DS 3 and DS<3 did not differ (p=0.73) (s. Figure SM2)

Figure SM2: Outcomes of patients with metabolic CR according to Deauville Score. A. Progression-free survival; B. Overall-survival
